# Management of zygomatic fractures using different surgical approaches

**DOI:** 10.6026/973206300191371

**Published:** 2023-12-31

**Authors:** Bibhu Prasad Mishra, Abhishek Harish, Alpha Mary Mathew, Asutosh Pradhan, Susmit Sneha, VijayaLaxmi Murty, Ramanpal Singh Makkad

**Affiliations:** 1 Department of Oral and Maxillofacial Surgery, Hi-tech Dental College and Hospital, Bhubaneshwar, Odisha, India; 2 Department of Oral and Maxillofacial Surgery, Government Medical College, Ambikapur, Chhattisgarh, India; 3 Department of Oral and Maxillofacial Surgery, Raipur, Chhattisgarh, India; 4 Department of Oral and Maxillofacial Surgery, Hi-tech Dental College and Hospital, Bhubaneshwar, Odisha, India; 5 Department of Oral Medicine and Radiology, Buddha Institute of Dental Sciences and Hospital, Patna, India; 6 Department of Prosthodontics, Crown and Bridge & Implantology, Raipur, Chhattisgarh, India; 7 Department of Oral Medicine and Radiology, New Horizon Dental College and Research Institute, Sakri, Bilaspur, Chhattisgarh, India

**Keywords:** Zygomatic complex fractures, treatment approach, surgical approach

## Abstract

Management of zygomatic complex fractures using closed reduction, two point open reduction with internal fixation (ORIF), closed reduction with three point ORIF and two point ORIF is of interest to dentist. 150 patients with zygomatic bone fractures between the ages of 14-60 years were included in the study. At final assessment, the percentage of stable condition was greater in closed reduction + two point ORIF and closed reduction + three point ORIF when compared to two point ORIF alone and three point ORIF alone and closed reduction alone. It was observed that stable condition was lowest in closed reduction alone. It was also observed that stable condition was lower in closed reduction + two point ORIF as compared to closed reduction + three point ORIF. It was also further noticed that stable condition was lower in two point ORIF alone as compared to three point ORIF alone. The treatment approach involving closed reduction and three point ORIF had better outcomes for management of zygomatic complex fractures.

## Background:

Zygomatic bone's structural design enables it to sustain impacts with significant force without breaking. Zygomatic bone separates from neighboring bone at or around the suture lines as a result of such strong stresses [1, 2,3]. It can become detached from its four joints, leading to a fracture of the zygomatic complex, zygomatico-maxillary complex or orbito-zygomatic. One of the most prevalent kinds of maxillofacial injuries that need to be treated is fractures of this complex [3,4]. Because of the intricate midface architecture, they are observed alone or in conjunction with other fractures in the face. Mouth opening may be reduced as a result of zygomatic bone fracture impingement over the coronoid process [5, 6]. A disturbance of the zygomatic alignment also affects the function, aesthetics, and psychology, impairing mandibular along with ocular function [7,8, 9]. Therefore, it is essential that zygomatic bone fracture be correctly diagnosed and appropriately addressed for both functional and aesthetic reasons [10,11]. An inadequate extension of the zygomatic body and consequent facial asymmetry are the outcome of skeletal repair of displaced fragments of zygomatic broken bones following inadequate fracture reduction and stabilization [12, 13]. The key to the quick repair of mid-facial fractures is an accurate estimation of the zygomatic bone's position with respect to the base of the skull at posterior position and the midface at anterior position. One of the biggest surgical challenges today is secondary reconstruction for orbito-zygomatico-maxillary complex abnormalities resulting from trauma [14,15]. For the cheeks to remain prominent and the face to remain typical in breadth, the zygomatic complex (ZC) must remain intact [16,17]. While zygomatic complex fractures (ZCF)with little or no displacement can often be treated non-surgically, whereas fractures resulting in functional or aesthetic impairments such as malocclusion, depression of the malar prominence, entrapment of extraocular muscles, diplopia and/or prohibited mouth opening frequently require surgical intervention [18, 19]. The optimal surgical strategy for reducing ZCF must minimize the risk of damage to face tissues, maximise the exposure of the broken segments, and guarantee both a good cosmetic and functional outcome [20, 21]. A frequently employed surgical method for the reduction of ZCF is the Gilles temporal approach. Nevertheless, there is a chance of facial nerve paralysis and a hairline scar connected with this surgical procedure [22,23]. Furthermore, in the event of an unstable ZCF, further exposure of the inferior border of orbital rim or the zygomaticofrontal junction is necessary for the implantation of mini-plates fixation [24,25,26, 27]. Keen first reported surgical management of zygomatic fractures using an intraoral surgical method in 1909, and since then, a number of investigations have reported on the course of treatment following open reduction of zygomatic complicated fractures using an intraoral surgical approach [12-23]. There are several surgical methods that have been reported for treating zygomatic complicated fractures. It is possible to achieve open reduction with surgical incisions using Keen's approach, the bicoronal scalp flap strategy, Gillies' approach or the more well-liked Dingman's approach [10-12]. The temporal approach is Gillies' method. One benefit of this surgery is that it is easy to execute and leaves no scars on the face. In the UK, zygomatic bone fractures are frequently treated with the Gillies temporal approach technique [14- 18]. Open reduction and internal fixation of zygoma fractures that are just displaced in an effort to identify the most straightforward technique for attaining post reduction stability [19- 22]. According to a report, the maximum stability was achieved with the three-point fixation fronto-zygomatic suture (FZS), inferior orbital rim, and zygomatico-maxillary buttress (ZMB) utilising either mini-plates alone or inter fragmentary wiring [27,28]. The only objective method for comparing different surgical techniques and their aftereffects is to use outcome assessments, which necessitate protocol management and extended follow-up [20-26]. With the exception of the treatment of solitary zygomatic arch fractures, the choice for open reduction and internal fixation of zygomatic fractures (ORIF) utilising three point fixations has increased in response to studies of subpar outcomes from two pint fixation technique [18-24]. There is no data on different combinations of closed reduction and ORIF approaches for management of ZCF. Therefore, it is of interest to compare closed reduction + two point ORIF, closed reduction + three point ORIF, two point ORIF alone, three point ORIF alone and closed reduction.

## Methods and Materials:

For the management of zygomatic bone fractures, 150 healthy individuals were scheduled. Patients who were split up into five therapy groups at random (Table 1). Patients with zygomatic bone fractures between the ages of 14 and 60 were included in the study. Patients with zygomatic bone fractures displaced in different directions but older than 15 days were included in the study, as were those with laterally displaced fractures as identified by clinical and radiographic findings on CT scan. The study excluded individuals with zygomatic bone fractures and gunshot wounds, as well as those who were medically unable to undergo general anesthesia or surgery. For the reduction of the fractures, the intraoral Keens technique and the Gillies temporal method were applied. The infraorbital edge, the frontozygomatic suture (FZS) and the zygomatico maxillary buttress (ZMB) region, and were the locations of fixation. The ZMB area and FZS were fixed in patients using the two-point fixation approach, whereas the infraorbital border, (ZMB) area and the FZS were fixed in patients using the three-point fixation technique. The type of therapy being used at the moment of the patient evaluation was unknown to the observer. The randomization procedure was concealed from the treating surgeons. Since the patients were aware that the purpose of the study was to compare the effects of two and three point fixation techniques on malar height as well as vertical dystopia, they were not blinded.

## Methods of reduction:

The Gillies Temporal Approach involves making a 2.5 cm incision in the temporal portion of the scalp's hear-bearing area, tilted at a 45° angle to the zygomatic arch. By inserting the Rowe zygoma elevator between the fascia and the temporalis muscle, fracture risk is decreased. The Keens technique involves making a little incision in the mucobuccal fold, right below the maxilla's zygomatic buttress, measuring about 1 cm. In order to prevent penetrating the fat pad in the temporal region, the elevator is passed upward behind the broken bone while keeping tight contact with the bone. Reduction is accomplished by pushing the bone outward and upward; as the bone is replaced, there may be a popping sound.

## Methods of fixation:

A variety of conventional incisions were used to reveal the fracture locations. A lateral eyebrow incision or an upper lid blephroplasty incision was used to reach the frontozygomatic suture in individuals who underwent the two point fixation procedure. By making an intraoral buccal sulcus incision, the ZMB was made visible. Subciliary incision or transconjunctival technique was used to achieve greater exposure of the infraorbital rim in patients who had undergone three point fixations. The FZS and the ZMB region were fixed using 1.5 mm miniplates, while the infraorbital boundary was fixed using 0.9 mm microplates. Patients who underwent two-point fixation had their fixation done at the FZS and ZMB area, whereas patients who underwent three-point fixation had their fixation done at the ZMB region, infra orbital boundary, and FZS.

## Assessments of outcomes:

All patients underwent a comprehensive preoperative examination and investigation with Caldwell's posterior-anterior view and Waters' perspective and CT scan. Prior to surgery, the patient's vertex view was used to measure the patient's malar height using a vernier calliper to compare the fractured site to the normal site. One reference point (the point where the intercanthal line and the midsagittal line intersect) and second point was chosen at the peak height of malar region from the patient's vertex view. The distance between these two points was measured both before and after surgery. Preoperative and postoperative measurements of vertical dystopia were made using a tracing paper to delineate the infraorbital boundary on a scale on Waters view, and a difference in the level of bony orbits demonstrated by palpation and comparison with the normal side. Evaluation of malar height and vertical dystopia was carried out in all study participants at first week, third week and sixth week of follow up. There was final assessment at 3 month follow up regarding stability and instability of reduced and fixed ZCF.

## Statistical analysis:

SPSS version 14.0 was used to analyse the data. Age, vertical dystopia, and Malar height were quantitative variables that were expressed as Mean ± SD. t test was utilised to compare the two groups. P-value (p<0.05) was considered statistically significant difference.

## Results:

Males were more prone to zygomatic fracture in all categories. Most of the study participants were in fourth decade of life in all categories. The time duration was greater in closed reduction + two point ORIF and closed reduction + three point ORIF as compared to Two point ORIF alone, Three point ORIF alone and closed reduction (Table 2). The malar height was comparable in all categories at first week follow up. At 3rd week and 6th week follow up, the malar height was greater in Closed reduction + two point ORIF and Closed reduction three point ORIF when compared to two point ORIF lone and three point ORIF alone and closed reduction. It was observed that malar height was lowest in closed reduction alone. It was also observed that malar height was lower in closed reduction + two point ORIF as compared to closed reduction + three point ORIF. It was also further noticed that malar height was lower in two point ORIF alone as compared to three point ORIF. The findings were statistically significant at 6th week follow up with p=0.04 (Table 3). The vertical dystopia was comparable in all categories at first week follow up. At 3rd week and 6th week follow up, the vertical dystopia was lower in Closed reduction + two point ORIF and Closed reduction + three point ORIF when compared to two point ORIF alone and three point ORIF alone and closed reduction. It was observed that vertical dystopia was greatest in closed reduction alone. It was also observed that vertical dystopia was greater in closed reduction + two point ORIF as compared to closed reduction + three point ORIF. It was also further noticed that vertical dystopia was greater in two point ORIF alone as compared to three point ORIF. The findings were statistically significant at both 3rd week (p=0.001) and 6th week follow up with p=0.000 (Table 4). At final assessment, percentage of stable condition was greater in closed reduction + two point ORIF and closed reduction + three point ORIF when compared to two point ORIF alone and three point ORIF alone and closed reduction alone. It was observed that stable condition was lowest in closed reduction alone. It was also observed that stable condition was lower in closed reduction + two point ORIF as compared to closed reduction + three point ORIF. It was also further noticed that stable condition was lower in two point ORIF alone as compared to three point ORIF alone (Table 5).

## Discussion:

Some studies has shown results similar to our study showing reduced malar height in two point fixation as compared to three point fixation [26,27]. However there were some studies also that found no significant difference in malar height in two point fixation and three point fixation [12- 21]. Because of its structural makeup, zygomatic bones can withstand hits with considerable force without fracturing. Such high strains cause the zygomatic bone to split off from the surrounding bone at or around the suture lines [12-15]. A fracture of the orbito-zygomatic, zygomatico-maxillary, or zygomatic complex can result from it breaking away from its four joints. Fractures of this complex are among the most common types of maxillofacial injuries that require medical attention [16-18]. They can be seen alone or in combination with other facial fractures due to the complex midface architecture. The impingement of a fractured zygomatic bone over the coronoid process may cause a reduction in mouth opening. In addition to compromising ocular function, a disruption of the zygomatic alignment also impacts function, aesthetics, and psychology [13- 17]. For both functional and cosmetic reasons, it is crucial that zygomatic bone fractures be accurately diagnosed and treated. Skeletal restoration of displaced pieces of zygomatic shattered bones after insufficient fracture reduction and stabilization results in an inadequate zygomatic body extension and ensuing facial asymmetry [18- 20]. Accurately estimating the zygomatic bone's position in relation to the midface at anterior and the base of the skull at posterior is crucial for the prompt treatment of mid-facial fractures [21- 24]. Secondary reconstruction for orbito-zygomatico-maxillary complex anomalies resulting from trauma is one of the largest surgical problems of the modern era [12-18]. Some studies has shown results similar to our study showing increased vertical dystopia in two point fixation as compared to three point fixation[27,28]. However there were some studies also that found no significant difference in vertical dystopia in two point fixation and three point fixation [16- 23]. The zygomatic complex (ZC) has to hold together for the cheeks to continue being prominent and the face to continue being normal in width [14-18]. While fractures causing functional or cosmetic impairments like malocclusion, depression of the malar prominence, entrapment of extraocular muscles, diplopia, and/or forbidden mouth opening often necessitate surgical intervention, ZCF with little to no displacement can often be treated non-surgically [17-24]. A favorable cosmetic and functional outcome, maximum exposure of the fractured segments, and little risk of harm to facial tissues are all necessary components of an ideal surgical strategy for minimizing ZCF [17-23]. A surgical technique that is often used to reduce ZCF is the Gilles temporal approach. However, this surgical approach carries a risk of hairline scarring and facial nerve paralysis. Moreover, additional exposure of the zygomaticofrontal junction or the inferior border of the orbital rim is required for the implantation of mini-plates fixation in the case of an unstable ZCF [21- 27]. After open reduction of complex zygomatic fractures using an intraoral surgical technique, several investigations have reported on the course of treatment [12-18]. The results of our study are in accordance to findings of previous study showing more number of cases with stable outcome in three point fixation [27,28]. However some studies showed similar results for both techniques [12-18]. Zygomatic complex fractures have been reported to be treated surgically using a variety of techniques [15- 21].Using Keen's approach, the bicoronal scalp flap strategy, Gillies' approach, or the more popular Dingman's approach, open reduction can be accomplished with surgical incisions. Gillies' approach is the temporal approach [13-17]. This operation has the advantage of being simple to perform and leaving no facial scars. The Gillies temporal approach technique is a common treatment for zygomatic bone fractures [12-8]. Just displaced zygoma fractures should be treated with open reduction and internal fixation in an attempt to determine the simplest method for achieving post reduction stability. A paper states that the three-point fixation (FZS, inferior orbital rim, and ZMB) using either miniplates alone or interfragmentary wire yielded the highest level of stability [27,28].

## Conclusion:

The treatment approach involving closed reduction and three point ORIF had better outcomes for management of zygomatic complex fractures.

## Figures and Tables

**Table 1:** Distribution of Study participants

	**Treatment approach**	**Number**
Category 1	Closed reduction alone	30
Category 2	Closed reduction with two point ORIF	30
Category 3	Closed reduction with three point ORIF	30
Category 4	Two point ORIF alone	30
Category 5	Three point ORIF alone	30
ORIF = open reduction and internal fixation

**Table 2:** Demographic details of study participants

	**Male: Female**	**Mean age (years ± SD)**	**Time duration (min)**
Closed reduction alone	5.23: 1	35.23	34.33
Closed reduction with two point ORIF	5.12:1	34.12	120.21
Closed reduction with three point ORIF	5.34:1	35.24	121.34
Two point ORIF alone	5.14:1	36.17	98.22
Three point ORIF alone	5.17:1	35.67	97.32
ORIF = open reduction and internal fixation

**Table 3:** Malar Height (mm) ± SDat 1st week, 3rd week and 6th week post operatively

	**First week**	**3rd Week**	**6th week**
Closed reduction (CR) alone	67.12± 1.13	65.34±3.21	65.14± 1.24
Closed reduction with two point ORIF	72.40 ± 5.42	70.24 ± 5.74	69.94 ± 3.62
Closed reduction with three point ORIF	72.24 ± 4.36	71.40 ± 5.84	71.23 ± 3.76
Two point ORIF alone	70.29 ± 4.31	68.13 ± 4.63	67.83 ± 3.62
Three point ORIF alone	70.13 ± 4.36	69.29 ± 4.73	69.4 ± 4.87
P value	0.804	0.06	0.04*
ORIF = open reduction and internal fixation; *indicates statistically significant difference

**Table 4:** Vertical Dystopia (mm) ± SD at 1st week, 3rd week and 6th week post operatively

	**First week**	**3rd Week**	**6th week**
Closed reduction alone	3.24±0.21	3.87± 0.19	4.34±0.01
Closed reduction with two point ORIF	2.34 ± 0.08	2.66 ± 0.92	3.24 ± 0.05
Closed reduction with three point ORIF	2.14 ± 0.39	2.28 ± 1.05	2.35 ± 0.02
Two point ORIF alone	2.95 ± 0.79	3.97 ± 0.92	4.13 ± 0.04
Three point ORIF alone	2.97 ± 0.97	3.17 ± 1.05	3.36 ± 0.02
P value	0.897	0.001*	0.0001*
ORIF = open reduction and internal fixation; *indicates statistically significant difference

**Table 5:** Final Evaluation at 3 month follow up

	**Stable**		**Unstable**	
	**Number**	**Percentage**	**Number**	**Percentage**
Closed reduction alone	10	33.34	20	66.66
Closed reduction with two point ORIF	14	46.67	16	53.33
Closed reduction with three point ORIF	27	90	3	10
Two point ORIF alone	12	40	18	60
Three point ORIF alone	25	83.34	5	16.67
ORIF = open reduction and internal fixation
